# Olfactomedin 4 Serves as a Marker for Disease Severity in Pediatric Respiratory Syncytial Virus (RSV) Infection

**DOI:** 10.1371/journal.pone.0131927

**Published:** 2015-07-10

**Authors:** H. K. Brand, I. M. L. Ahout, D. de Ridder, A. van Diepen, Y. Li, M. Zaalberg, A. Andeweg, N. Roeleveld, R. de Groot, A. Warris, P. W. M. Hermans, G. Ferwerda, F. J. T. Staal

**Affiliations:** 1 Laboratory of Pediatric Infectious Diseases, Department of Pediatrics, Radboud university medical center, Nijmegen, The Netherlands; 2 Radboud Institute for Molecular Life Sciences, Radboud university medical center, Nijmegen, The Netherlands; 3 Delft Bioinformatics Lab, Faculty of Electrical Engineering, Mathematics and Computer Science, Delft University of Technology, Delft, The Netherlands; 4 Department of Bioinformatics, Erasmus University Medical Center, Rotterdam, The Netherlands; 5 Department of Virology, Erasmus University Medical Center, Rotterdam, The Netherlands; 6 Department for Health Evidence, Radboud university medical center, Nijmegen, the Netherlands; 7 Department of Immunohematology and Blood Transfusion, Leiden University Medical Center, Leiden, The Netherlands; 8 Department of Immunology, Erasmus University Medical Center, Rotterdam, The Netherlands; Kliniken der Stadt Köln gGmbH, GERMANY

## Abstract

**Background:**

Respiratory viral infections follow an unpredictable clinical course in young children ranging from a common cold to respiratory failure. The transition from mild to severe disease occurs rapidly and is difficult to predict. The pathophysiology underlying disease severity has remained elusive. There is an urgent need to better understand the immune response in this disease to come up with biomarkers that may aid clinical decision making.

**Methods:**

In a prospective study, flow cytometric and genome-wide gene expression analyses were performed on blood samples of 26 children with a diagnosis of severe, moderate or mild Respiratory Syncytial Virus (RSV) infection. Differentially expressed genes were validated using Q-PCR in a second cohort of 80 children during three consecutive winter seasons. FACS analyses were also performed in the second cohort and on recovery samples of severe cases in the first cohort.

**Results:**

Severe RSV infection was associated with a transient but marked decrease in CD4+ T, CD8+ T, and NK cells in peripheral blood. Gene expression analyses in both cohorts identified Olfactomedin4 (OLFM4) as a fully discriminative marker between children with mild and severe RSV infection, giving a PAM cross-validation error of 0%. Patients with an OLFM4 gene expression level above -7.5 were 6 times more likely to develop severe disease, after correction for age at hospitalization and gestational age.

**Conclusion:**

By combining genome-wide expression profiling of blood cell subsets with clinically well-annotated samples, OLFM4 was identified as a biomarker for severity of pediatric RSV infection.

## Introduction

Respiratory viral infections are an important cause of hospitalization among children younger than 5 years of age. Human Respiratory Syncytial Virus (RSV) is the most common (40–85%) identified virus in infants hospitalized for respiratory infections during winter epidemics, with hospitalization rates between 1 and 2% [1–6]. Clinical manifestations range from common colds to severe lower respiratory tract infections requiring mechanical ventilation. Risk factors for a severe course are known, but the majority of patients admitted to an Intensive Care Unit were previously healthy [7–9]. Since transition from mild to severe disease can occur within hours, one of the key challenges for clinicians is to differentiate children who need hospitalization for supportive care from those who can safely be discharged. Currently, young infants with mild bronchiolitis, especially those younger than 12 weeks of age, are often admitted to a hospital since they have an increased risk of severe disease. However, up to 35% of children hospitalized with bronchiolitis do not receive any supportive intervention [10]. Conversely, it is crucial to avoid early discharge of those children who may experience clinical deterioration. Among children sent home with the diagnosis bronchiolitis, 4.6–6.8% require hospitalization later on [11, 12].

Much research has been done on the immune response against RSV in humans. Several reports suggested an important role for the innate immune system, while others found an inadequate adaptive immune response especially in young children and in individuals who present with a severe clinical picture [13]. The uncertainty in the nature of the immune response against RSV is reflected in the unpredictable clinical course of the infection as well as in the difficulty of developing an adequate vaccine. We as well as others previously reported that T lymphocytes can be markedly decreased in the more severe cases of the disease. We reported that in severe cases both CD4 and CD8 T cell numbers, as well as NK cells were reduced in peripheral blood [14]. However, it remains unclear whether this indicates an inadequate immune response against RSV, for instance by massive apoptosis or decreased production of T cells, or that peripheral blood poorly reflects an ongoing immune response that might be very active.

The detection and application of biomarkers to assess severity of viral lower respiratory tract infections, in particular RSV infection, may assist clinicians in the prediction of severe disease in children with bronchiolitis and may help to reduce the number of unnecessary hospitalizations or clinical deterioration after discharge. Furthermore, markers for disease severity are important research tools to study effects of interventions by new therapies or to stratify patients by disease severity [15, 16]. Several studies have shown that transcriptional analysis of peripheral blood cells may be used to define different etiologies of disease and disease outcomes [17–20]. In a seminal proof-of-concept study, Ramilo *et al*. (2007) compared the transcriptional profiles of PBMCs of children with infectious diseases and identified a set of genes that can separate influenza A infections from bacterial infections (*Staphylococcus aureus*, *Escherichia coli* and *Streptococcus pneumoniae*) [20]. Recently this group also reported that these transcriptome profiles also contained information regarding viral etiology (influenza, rhino virus and RSV) and the course of disease [21].

This study was initiated to obtain insight into the changes occurring in adaptive and innate immune cells during RSV infection and to identify possible biomarkers of disease severity. To identify transcriptional biomarkers to separate mild from severe disease, genome-wide gene expression analyses were performed on blood samples of 26 children with a diagnosis of severe, moderate or mild RSV infection in two winter seasons. A validation cohort of 80 children spanning three other consecutive winter seasons by flow cytometry and Q-PCR was used to validate various candidate biomarkers.

## Material and Methods

### Study design

In this prospective cohort study, 3 ml of Sodiumheperanized blood and nasopharyngeal samples were obtained from two cohorts of patients with RSV bronchiolitis within 24 hours after first contact with the hospital. Medical history, demographic data, and clinical assessments were collected from questionnaires and medical records. Exclusion criteria were corticosteroid use in past 48 hours, congenital significant heart or lung disease and immunodeficiency, Presence of 15 different viral pathogens was tested by multiplex RT-PCR on nasopharyngeal samples as previously described [22]. Patients were classified retrospectively into three groups based on severity of disease. The mild group included children without hypoxia or severe feeding problems. The moderate group included children requiring hospitalization for supplemental oxygen (oxygen saturations <93%) and/or nasogastric feeding. Children requiring mechanical ventilation were included in the severe group. Recovery samples were obtained 4–6 weeks after acute infection from children with moderate and severe disease. The first cohort consisted of 26 patients with RSV infections, divided into mild (n = 9), moderate (n = 9) and severe (n = 8) disease. From all moderate and severe diseased patients recovery samples were obtained. This cohort was used for micro-array analysis and initial qPCR validation of genes of interest. The second cohort comprised 80 children with viral lower respiratory tract infections both RSV positive and negative, and was also divided into three groups: mild (n = 14), moderate (n = 42) and severe (n = 24). This cohort was meant for validation purposes. All subjects were recruited at two hospitals in Nijmegen, the Canisius Wilhelmina Hospital and the Radboud university medical center, the Netherlands. The study protocols were approved by the institutional review board (Commissie Mensgebonden Onderzoek: Regional Committee on Research involving Human Subjects Arnhem-Nijmegen, The Netherlands) and were conducted in accordance with the principles of the Declaration of Helsinki. Written informed consent was obtained from the parents of all children.

### RNA isolation and microarray gene expression analyses

Peripheral blood mononuclear cells (PBMCs) were isolated by density gradient centrifugation (Lymphoprep, Axis Shield, Norway), counted and subsequently stored in Trizol reagent (Invitrogen, The Netherlands) at -80°C in the same laboratory by the same team for both cohorts. RNA from PBMC was extracted using Trizol (Invitrogen Life Technologies) according to the manufacturer’s protocol. Total RNA was isolated using the RNeasy Minikit (Qiagen). RNA integrity and quality was assessed using capillary electrophoresis [RNA 6000 Nano LabChip (Agilent)] on an Agilent Bioanalyzer 2100 system. RNA processing, target labeling and hybridization to gene expression arrays was performed by standard methods as described [23]. Biotin labeled cRNA was obtained using the One-Cycle Eukaryotic Target Labeling Assay (Affymetrix), after which 15 μg of fragmented, biotin labeled cRNA was hybridized to Affymetrix GeneChip Human Genome U133 plus 2.0 arrays according to standard Affymetrix protocol (Affymetrix Inc, Santa Clara, CA).

### Flow Cytometry

Immunophenotyping of cryopreserved PBMCs were performed after thawing. The following combinations of markers and fluorescent antibodies were used: CD14–FITC, CD16.56–phycoerythrin, CD3–peridinin chlorophyll protein, CD19–allophycocyanin, CD4–phycoerythrin–Cy7, and CD8–allophycocyanin–Cy7 (all Beckman Coulter, Miami, FL). Samples were acquired immediately after staining on a BD FACSCanto (Becton Dickinson, Heidelberg, Germany) and analyzed using flow cytometry analysis software (FlowJo analyses 7.6, Three Star, Ashland, OR). The following subsets were defined: CD4+ T cells (CD4+CD3+CD8-), CD8 T cells (CD8+CD4-CD3+), NK cells (CD3-CD56+), B cells (CD45+CD19+) and monocytes (CD14+).

### Data analysis

Quality control analyses were performed as previously described [23, 24]. Scanned images were inspected for artifacts, percentage of calls present (<25%) and controls of RNA degradation. This led to some arrays being discarded. On each remaining array, probes labelled outliers by the Affymetrix scanning software and overexposed probes (with maximum PM intensity level >63.000) were removed. Subsequently, probesets with less than 8 probes remaining were discarded. For each comparison, robust multichip analysis (RMA) was used for background removal, quantile normalization of probe intensity levels and probe set summarization. The resulting values were log_2_-transformed for further analysis, giving probeset expression levels between 0 and 16. We then selected only those probesets that showed at least a two-fold difference (up or down) on a minimum of two arrays with respect to the median expression over all arrays in that particular comparison [24, 25]. Finally, Significance Analysis of Microarrays (SAM, [26]) was applied to find differentially expressed probesets with a significance level of *q* <0.05. To select only biologically relevant changes, we demanded additionally that the absolute expression level was larger than log_2_(200) and that the absolute difference between groups was larger than 2 fold.

In a subsequent supervised analysis, we trained a PAM classifier (“Prediction Analysis of Microarrays” [27]), attempting to find a minimum number of discriminative genes that yielded an optimal cross-validation error (i.e. the predicted test error). For visualization purposes, samples were clustered based on selected probesets by complete linkage hierarchical clustering with 1-correlation as a distance measure, using the Matlab Bioinformatics toolbox (Mathworks, Natick, MA). The original and processed data were deposited in the NCBI Gene Expression Omnibus (GEO; http://www.ncbi.nlm.nih.gov/geo; GSE69606). All microarray experiments were performed according to the MIAME guidelines.

### RT-PCR

Real-time quantitative PCR was used to measure the expression of genes of interest. Initial validation of gene expression of OLFM4 detected in the first cohort was performed with SYBR Green PCR Mastermix (Applied Biosystems; P/N 4367659) with forward 5`- atcaaaacacccctgtcgtc- 3`and reverse 5`- gctgatgttcaccacaccac-3`primers for OLFM4. Actin was used as a reference gene with forward primer 5`- cgtcacacttcatgatggagttg-3`and reverse primer 5`-cttccttcctgggcatgga-3`. After validation of the microarray, the second cohort was analyzed with commercially available Taqman primers (OLFM4 Hs00360669_m1 and GAPDH Hs99999905_m1). All samples were run for 40 cycles in duplicate on an Applied Biosystems 7500 Fast Real-Time PCR System. Ct values of OLFM4 were normalized against the reference gene GAPDH.

### OLFM4 plasma measurement

OLFM4 concentrations were measured in randomly selected plasma samples of 49 patients from the validation cohort by a commercial ELISA kit (E90162Hu, Uscn Live Science Inc., China) according to the instructions of the manufacturer.

### Published microarray data mining

A data mining search was performed in NCBI GEO and in EBI Arrayexpress, online databases with datasets and profiles of previously performed microarray studies to validate our results [28, 29]. Terms for searching were: *OLFM4*, *Affymetrix*, *whole blood children*, *RSV and/or homo sapiens*. More than 90 microarray studies were found. Based on the population (children/infants), sample size, disease type and available information per sample, 18 studies were selected. From the series matrix files, the results were log transformed and OLFM4 gene expression was selected and analyzed to gain insight in its behavior in different disease states and ages.

### Statistics

The distributions of categorical variables are presented as percentages per category. Numerical variables are reported as means with standard deviation (SD) or medians with interquartile ranges (IQR) depending on whether or not the variables were normally distributed (Kolmogorov-Smirnov’s test, *p*>0.05). To determine whether OLMF4 was independently associated with receiving mechanical ventilation, multivariable log-binomial regression analyses were performed in the validation cohort resulting in adjusted Relative Risks (RR) [30]. Analysis were performed with SPSS v21 and graphpad v5.

## Results

We previously reported that RSV infection, especially in severe cases, was associated with lymphopenia. This was not only visible in NK and CD8+ T cells, known to be directly involved in anti-viral immunity, but surprisingly also in CD4+ T cells, whereas B cells were unaffected [14]. In the current study, we analyzed recovery samples of 6 severe patients after clearance of the infection (on average 4 weeks after discharge) and found that the numbers of NK cells as well as CD4+ and CD8+ T cells had returned to normal, indicating that the lymphopenia was transient (S1 Fig).

### Microarray analyses point to OLFM4 as a marker gene to classify disease severity

**Fig 1:**
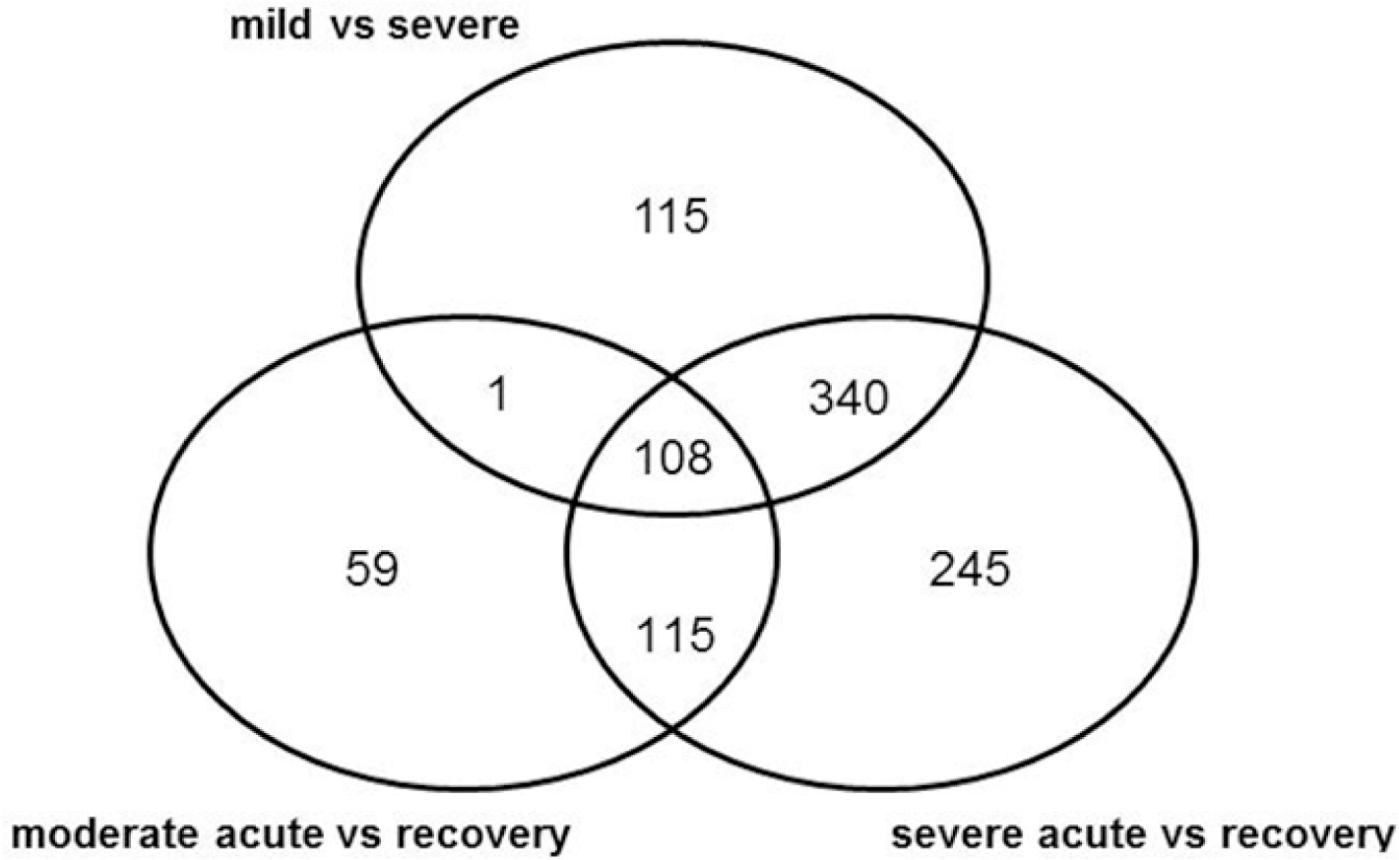
Venn diagram with differentially expressed genes between groups. Differentially expressed genes (q <0.05; >2 fold difference; absolute expression value >200) in patients with RSV infections comparing patients with mild vs severe disease and during acute infection vs recovery in patients with moderate and severe disease.

Patients used for microarray analysis with a clinical diagnosis of mild disease were older at time of admission to hospital than those with severe disease. The length of stay in hospital increased with increasing disease severity. No statistically significant differences were seen in gender, number of premature infants and duration of symptoms (Table 1). The microarray analysis of PBMC of children with mild versus severe disease showed that 564 probesets were expressed differentially (428 upregulated and 136 downregulated genes) under conditions as described in material and methods (q <0.05; >2 fold difference; absolute expression value > log_2_(200). As biomarkers should discriminate between non-disease and disease, the genes expressed differentially in children with mild versus severe disease as well as during acute severe RSV infection versus recovery were selected. The analysis of paired acute and recovery samples of children with severe RSV infection resulted in 808 differentially expressed probesets (647 upregulated and 161 downregulated). Of these 808 probesets, 448 showed overlap with the 564 probesets in the comparison of mild versus severe disease, 365 genes being upregulated and 83 genes being downregulated (Fig 1 and S2 Fig). Table 2 shows the top 25 of up- and downregulated genes, of which *Olfactomedin 4* (OLFM4) was the most upregulated gene with a factor of over 40 fold. Since children in the severe group were younger compared to those with mild disease, a paired age-matched subanalysis was performed among 7 severe patients versus 7 patients with mild or moderate disease. This analysis resulted in 287 differentially expressed probesets, all upregulated. The gene list of upregulated probesets did not differ substantially from the main analysis. A supervised analysis (PAM) also identified OLFM4 as a fully discriminative marker between children with mild and severe RSV infection, giving a cross-validation error of 0%. As both SAM and PAM analyses revealed OLFM4 as a potentially important marker for disease severity in children with RSV infection and OLFM4 has-to the best of our knowledge- not been associated with respiratory tract infections before, this gene was chosen for further analysis. Interestingly, there was no marked upregulation of apoptosis genes in the severe group, indicating that the observed lymphopenia was not caused by increased apoptosis.

**Table 1:** Patient characteristics. Values are given in numbers (percentages) and median with inter quartile range (IQR). *P*-values are based on Kruskall Wallis tests, followed by Mann Whitney U tests for individual comparisons:

	Mild (N = 9)	Moderate (N = 9)	Severe (N = 8)	*p*-value
**Age (months)**	8.7 [3.6–9.3]	1.9 [1.5–8.3]	2.4 [1.1–4.9]	NS
**Gender (male)**	6 (67%)	8 (89%)	6 (75%)	NS
**Gestational age (wks)**	40 [36.9–41.0]	38.6 [37.2–40.0]	35.1 [33.1–39.8]	NS
**Length of stay (days)**	0 [0–3]	5 [2–9]	13 [6.3–19.8]	*p*<0.001*

*mild vs moderate *p*<0.01, moderate vs severe *p*<0.05, mild vs severe *p*<0.001

**Table 2:** Top 25 up- and downregulated genes differentially expressed in PBMCs during severe RSV infection. Genes that showed overlap in both the comparisons mild vs severe disease and severe vs recovery samples are shown.

Upregulated genes	Fold difference mild vs severe	Fold change severe vs recovery	Downregulated genes	Fold difference mild vs severe	Fold change severe vs recovery
**OLFM4**	49.7	43.2	**GNLY**	-4.8	-4.0
**MMP8**	24.2	37.2	**GZMH**	-4.5	-3.2
**MMP8**	24.1	33.6	**GNLY**	-4.5	-3.7
**CAECAM8**	18.4	19.3	**FGFBR2**	-4.5	-5.1
**ARG1**	16.9	24.2	**TRAC**	-3.9	-2.9
**ANXA3**	16.6	16.2	**KLRF1**	-3.8	-3.3
**DEFA4**	15.1	16.1	**LGALS2**	-3.7	-2.0
**CA1**	14.9	17.5	**KLRC1**	-3.7	-3.2
**CHI3L1**	13.9	11.0	**KLRD1**	-3.7	-3.0
**LTF**	13.0	12.6	**TRAC**	-3.4	-2.8
**SELENBP1**	12.8	15.8	**THOC4**	-3.4	-3.1
**CRISP3**	12.3	12.3	**GZMB**	-3.4	-2.3
**ELANE**	11.2	10.6	**KLRD1**	-3.4	-2.7
**HP**	11.0	11.9	**IGHM**	-3.3	-2.0
**CEACAM6**	11.0	10.1	**GZMK**	-3.3	-2.4
**HBM**	10.5	26.0	**PRF1**	-3.2	-2.2
**CHI3L1**	10.3	9.2	**TRAC**	-3.1	-2.3
**MPO**	10.2	9.9	**ITPKB**	-3.0	-3.5
**ALAS2**	10.2	21.7	**SH2D1B**	-3.0	-2.6
**IL1R2**	10.2	10.5	**SPON2**	-3.0	-2.8
**EPB42**	10.1	15.3	**PRF1**	-3.0	-2.4
**CEACAM6**	10.1	9.7	**TGFBR3**	-2.9	-2.4
**LCN2**	10.1	10.3	**FCER1A**	-2.9	-3.9
**MPO**	9.8	9.9	**KLRB1**	-2.9	-3.9
**MMP9**	9.8	11.6	**GPR56**	-2.9	-2.9

### Validation of microarray findings by qPCR

**Fig 2:**
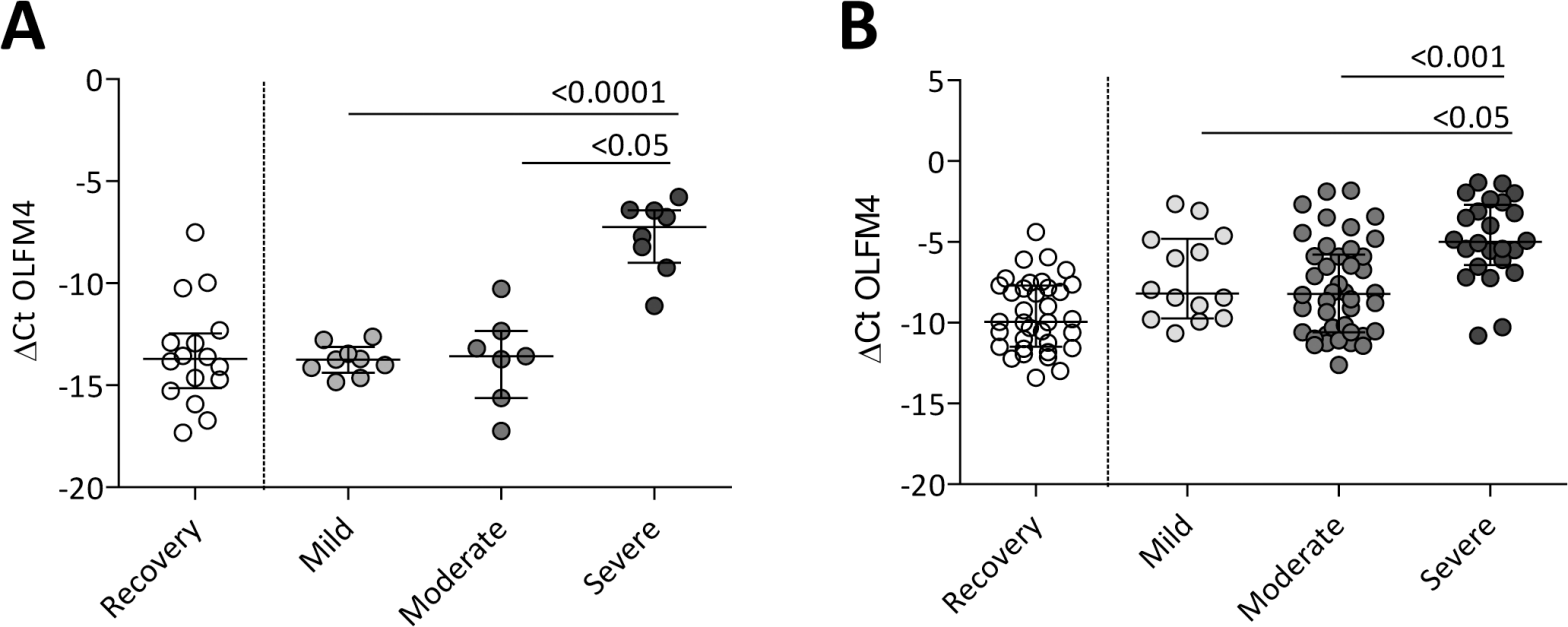
OLFM4 gene expression values of patients from the micro-array and validation cohort. OLFM4 gene expression levels were significantly higher in patients with severe disease compared to those with mild and moderate disease in the micro-array cohort (Panel A) and an independent validation cohort (Panel B). Expression levels are presented as ΔCt and median with inter quartile range (IQR). Statistics were performed by Kruskall Wallis tests, followed by Mann Whitney U tests for individual comparisons.

To confirm our findings in the microarray analyses, qPCR was performed for OLFM4 in PBMCs of the same patients. One patient in the moderate group was excluded since insufficient material was left. OLFM4 expression was statistically significantly higher in patients with severe disease compared to those with mild and moderate disease (Fig 2A).

### OLFM4 gene expression in PBMCs is increased during acute viral respiratory infection and correlates with disease severity in a validation cohort

The validation cohort consisted of 80 children with viral lower respiratory tract infections, among which 47 had a confirmed RSV infection. This cohort reflects the patients presenting in a paediatric ward during respiratory season prior to viral diagnostics, therefore both RSV positive and negative patients were analysed. The characteristics of these patients differed from those of the patients in the discovery cohort, especially for age, gender, preterm birth, duration of symptoms. (S1 Table). In total, 115 PBMC samples were available for qPCR analysis, subdivided in 80 acute and 35 recovery samples. OLFM4 gene expression levels during acute infection were higher compared to those obtained after recovery (*p*<0.001). In agreement with our microarray analyses, expression of OLFM4 in PBMCs was higher in patients with severe disease compared to those with mild and moderate disease (Fig 2B). For the confirmed RSV positive patients only, OLFM4 expression also served as a discriminating marker, similar to the full validation cohort (S3 Fig). Length of stay (LOS) in hospital, another measure for severity, was also correlated positively with gene expression levels of OLFM4 (*ρ* = 0.402, *p*<0.001).

### OLFM4 expression correlates with disease severity in PBMCs but not in plasma

**Fig 3:**
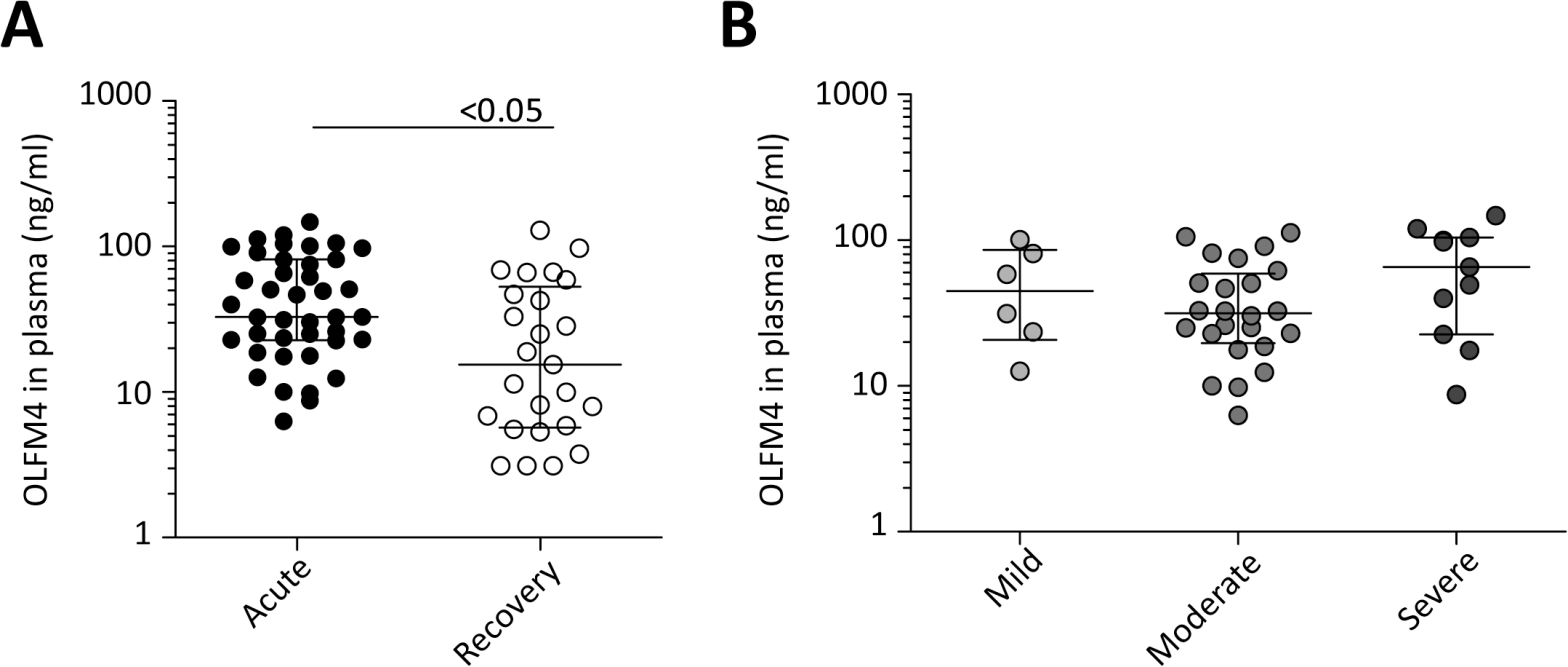
Plasma levels of OLFM4 in patients with viral RTI. OLFM4 plasma levels are statistically significantly higher during acute (n = 41) infections compared to recovery samples (n = 25) (Panel A). However, there are no statistically significant differences among the three severity groups (Panel B). Plasma levels (ng/ml) are presented as median with inter quartile range (IQR). Statistics were performed by Mann Whitney U tests for comparison acute vs recovery (*p*<0.05), and Kruskall Wallis tests for comparison mild vs moderate vs severe (*p* = 0.29).

Since biomarkers in plasma are more easily obtained and processing is less time-consuming, we measured protein levels of OLFM4 in plasma of 49 randomly selected patients of the validation cohort. Although OLFM4 plasma concentrations during acute infection were statistically significantly higher compared to those in recovery samples, no association with disease severity was observed (Fig 3). No correlation between protein levels and relative gene expression was found either (*ρ* = 0.270, *p* = 0.088).

### In a multivariable model OLFM4 gene expression is a statistically significant marker for severe disease

To determine the predictive value of OLFM4 in patient with acute viral bronchiolitis, both RSV positive and negative patients we performed a multivariable analysis. Relative OLMF4 gene expression, gender, gestational age, and age at time of hospital admission (in weeks) were included as determinant and potential confounders, respectively in a multivariable model for mechanical ventilation (Table 3).

**Table 3:** Multivariable analysis of the association between OLFM4 expression levels and the risk of getting mechanical ventilation. Values are given in numbers (percentages) and mean ± standard deviation. MV = mechanical ventilation, NS = not significant, OR = odds ratio, CI = confidence interval

	Characteristics			Univariate analysis N = 80	Multivariate analysis		
	MV (n = 24)	No MV (n = 56)	p-value	p-value	p-value	OR	(95% CI)
**Gender (male)**	12 (50%)	30 (54%)	NS	NS	-	-	-
**Age (weeks)**	1.22 [0.63–2.84]	3.95 [1.95–12.7]	<0.001	<0.01; OR 0.96	0.05	0.96	0.93–1.00
**OLFM4 expression**	-4.89 ± 2.53	-7.68 ±2.86	<0.001	4,71*10E-4; OR 1.43	**<0.01**	1.48	1.14–1.91
**Premature (<37wks)**	4 (17%)	11 (20%)	NS	NS	-	-	-
**Duration symptoms**	11.0 [10.0–13.0]	5.0 [2.0–4.0]	NS	NS	-	-	-

For OLMF4, a cut-off value of > -7.5 was chosen, which corresponds with an OLFM4 expression level greater than 0.5% that of GAPDH. The unadjusted RR of mechanical ventilation was 8.6 with a 95% confidence interval (CI) of 2.2–34.0. After adjustment for age and gestational age, the RR was 6.1 (95%CI: 1.5–24.4), which indicates that children with OLMF4 gene expression levels above -7.5 have a 6-fold increased risk of severe infection requiring mechanical ventilation (Table 4). Gender did not add substantially to the final model. Including OLMF4 gene expression in the model as a continuous variable resulted in an age and gestational age adjusted RR of 1.20 (95%CI: 1.04–1.38), meaning that the risk of receiving mechanical ventilation increased by 20% with every step increase in expression level (range -12.6 through -1.33).

**Table 4:** Relative risk of getting mechanical ventilation determent by OFFM4 gene expression. OR = odds ratio, CI = confidence interval

	Multivariate analysis	p-value	OR 95% (CI)	Relative risk
**OLFM4 expression> -7.5**	**<0.01**	**15.78**	1.93–46.57	8.7

## Discussion

In this study we demonstrated that OLFM4 gene expression in PBMC is a previously unidentified classifier for severe disease in children with viral lower respiratory infections. OLFM4 expression was significantly increased during acute viral respiratory infections compared to recovery samples. Moreover, an association was found between OLFM4 gene expression in PBMCs and disease severity; in a multivariable model OLFM4 showed its power as a significant marker for severe disease. Children with mechanical ventilation have almost 10 times more often an increased OLFM4 expression in PBMC (greater than 0.05% of the GAPDh levels). Therefore, OLFM4 fulfills the criteria as a biomarker for disease severity, in particular to discriminate mild from severe cases in young infants. For OLFM4 to formally be used as prognostic marker, a more extensive, prospective study will be required.

To the best of our knowledge, the OLFM4 gene has not been described in the context of viral respiratory infections. Although changes in cell populations occur during the acute phase of infection could be reflected in the gene expression profiles, it is remarkable that predominantly upregulated genes indicate severe disease, whereas only decreased cell populations are observed [14]. To validate the OLFM4 gene expression changes during infectious disease we data-mined other micro-array studies that described pediatric and adult patient cohorts. In a study by Ioannidis *et al*. (GSE 34205), we found that OLFM4 gene expression was higher in PBMCs obtained from patients with RSV (n = 51) or influenza virus (n = 27) infections compared to the gene expression in healthy infants (n = 22), *p*<0.01 and *p*<0.0001, respectively [31]. No differences were found in OLFM4 gene expression between children under or above three months of age with either infection by RSV or influenza. In contrast, two other studies did not observe upregulation of OLFM4 in PBMCs from children during infection by measles or rotavirus (GSE 5808 and 2729) [32, 33]. Zaas *et al*. performed microarrays on whole blood obtained from adult volunteers at baseline and at the peak of their symptoms after being experimentally infected with RSV, influenza or rhinovirus (GSE 17156) [34]. Although their data showed an upregulation of OLFM4 in RSV infected adults (*p* = 0.01), there were no differences in OLFM4 gene expression between the baseline and during symptomatic influenza or rhinovirus infections [34]. Data of Ramilo *et al*. (GSE 6269–1) showed a statistically significant upregulation of OLFM4 in children, aged 0–16 years, diagnosed with influenza virus or bacterial infections (*E*. *coli*, *S*. *aureus* or *S*. *pneumoniae*) compared to healthy controls [20]. In this cohort, children with *S*. *aureus* or *S*. *pneumoniae* infections had statistically significantly higher OLFM4 gene expression compared to influenza A infected patients [20]. Thus, other studies have also seen upregulation of OLFM4 expresion after bacterial or viral infection. However, none of the studies looked at disease severity.

OLFM4, also known as hGC-1 and GW112, was first cloned from G-CSF–stimulated human myeloid precursor cells and is mainly expressed in bone marrow, gastro-intestinal tract, prostate and pancreas [35]. Earlier studies have shown that OLFM4 is involved in multiple cellular functions e.g. cell growth, differentiation and apoptosis [27]. OLFM4 expression has been reported as one of several (prognostic) markers in oncology [27]. In addition, its involvement in the immune response to inflammation has been described. OLFM4 expression is upregulated in some inflammatory diseases, such as chronic inflammatory bowel diseases [36] and in *Helicobacter pylori*-infected patients [37]. Liu *et al*. showed an enhanced immune response and inflammation in OLFM4-/- mice upon *Helicobacter pylori* infection. Their results indicate that OLFM4 inhibits NOD1 and NOD2-mediated NF-κB activation, suggesting that OLFM4 plays an important role in regulating innate immune responses [38]. In another study, Liu *et al*. demonstrated that neutrophils from OLFM4 -/- mice have increased capability to kill *S*. *aureus* and *E*. *coli* and are more resistant to systemic sepsis [39]. These data suggest that OLFM4 may be an important regulator of host innate immunity against a broad array of bacterial infections. Data mining of gene expression profiling datasets (www.immgen.org) indicates that OLFM4 is besides being expressed in neutrophils, also highly expressed in Th1 cells. Therefore the increased OLFM4 expression seen in the severe subgroup may indicate an highly active ongoing Th1 response. In summary, OLFM4 was upregulated in several viral and bacterial infections in many (but not all) previously published studies investigated.

Although OLFM4 mRNA has been described to be selectively expressed in normal human myeloid lineage cells, OLFM4 protein concentrations have been measured in PBMCs, B-lymphocytes, neutrophils and monocytes [40]. This is in agreement with our results and those from the reanalyzed microarray studies, in which high and significantly different OLFM4 mRNA expression was found in PBMCs obtained from children with different severity of viral lower respiratory infections.

Clemmensen *et al*. showed that OLFM4 was present at protein level in only 20–25% of peripheral blood neutrophils, whereas mRNA for OLFM4 was present in all myelocytes and metamyelocytes, indicating post-transcriptional regulation as a basis for the heterogeneous expression of OLFM4 protein [41]. This could explain the observed differences in our study between the transcription levels and the plasma concentration of the protein.

The advantage of measuring markers in plasma is the ease of implementation, speed, reproducibility and standardization. However, innovative techniques enable rapid analysis of the expression of multiple genes at transcriptional level in the near future [42].

The severe CD4, CD8 and NK cell lymphopenia that we and others described before, can now be better explained. Together with the up regulation of activation markers on PBMC in the severe group and lack of apoptosis, the observed lymphopenia likely results from recruitment of T cells to the site of infection, i.e the lungs with an parent lower cell count in peripheral blood. In this respect peripheral blood may not reflect the situation in all parts of the body. OLFM4 is associated with Th1 responses, an active or even overaggressive Th1 response may underlie the severe clinical manifestations in this group. This is reminiscent of interpretations in the early RSV vaccine trails. Nevertheless, more accurate measurements at the site of infection will be needed to determine whether in the severe cases an inadequate adaptive immune response or a hyper responsive reaction (for instance by excessive production of cytokines) is responsible for the severe manifestations of RSV infection.

Notwithstanding this uncertainty, in this study we are the first to show that OLFM4 transcription is associated with severity of disease in children with viral lower respiratory tract infections, also after correcting for age. These results emphasize the role of OLFM4 in innate and adaptive immunity and encourages further research into the presence of OLFM4 in PBMCs and the pathogenesis of RSV infections. Moreover, it could lead to the development of a new diagnostic tool to predict a severe course of viral respiratory disease and aid the physician in clinical decisions.

## Supporting Information

S1 Fig Transient lymphopenia during severe RSV infection. Immune phenotyping with flowcytometry of circulating leukocytes of infants with severe RSV infection (n = 6) in the acute phase and after clearance of the infection (on average 4 weeks after discharge). The numbers of NK cells as well as CD4+ and CD8+ T cells return to normal, indicating that the lymphopenia was transient. Statistics were performed by paired students-t test, significance was set at *p*<0.05.
(TIF)

S2 Fig Discriminating mild disease from severe disease in children with RSV infection. 448 differentially expressed probesets were selected based on overlap in the comparison mild vs severe disease in RSV infected children and acute samples vs recovery samples of children with severe RSV infection. Samples were clustered based on these selected probesets by complete linkage hierarchical clustering with 1-correlation as a distance measure.
(TIF)

S3 Fig OLFM4 gene expression values in PBMCs from RSV+ infants during acute mild, moderate and severe viral lower RTI and after recovery. Expression levels are presented as ΔCt and median with inter quartile range (IQR). Statistics were performed by Kruskall Wallis tests (*p*<0.001), followed by Mann Whitney U tests for individual comparisons: mild vs moderate *p* = 0.36, moderate vs severe *p*<0.001, mild vs severe *p* = 0.51.
(TIF)

S1 Table Patient characteristics of validation cohort. Values are given in numbers (percentages) and median and inter quartile range (IQR * mild versus severe *p* = 0.05, moderate versus severe *p*<0.001 ** mild versus moderate and severe *p*<0.001, moderate versus severe *p*<0.001 *** mild versus moderate *p*<0.01, mild versus severe *p<*0.05.
(DOCX)
