# Supplementary figures and images for: Olfactomedin 4 Serves as a Marker for Disease Severity in Pediatric Respiratory Syncytial Virus (RSV) Infection
Source: PLoS One. 2015 Jul 10;10(7):e0131927. doi: 10.1371/journal.pone.0131927 (PMC4498630; doi:10.1371/journal.pone.0131927)

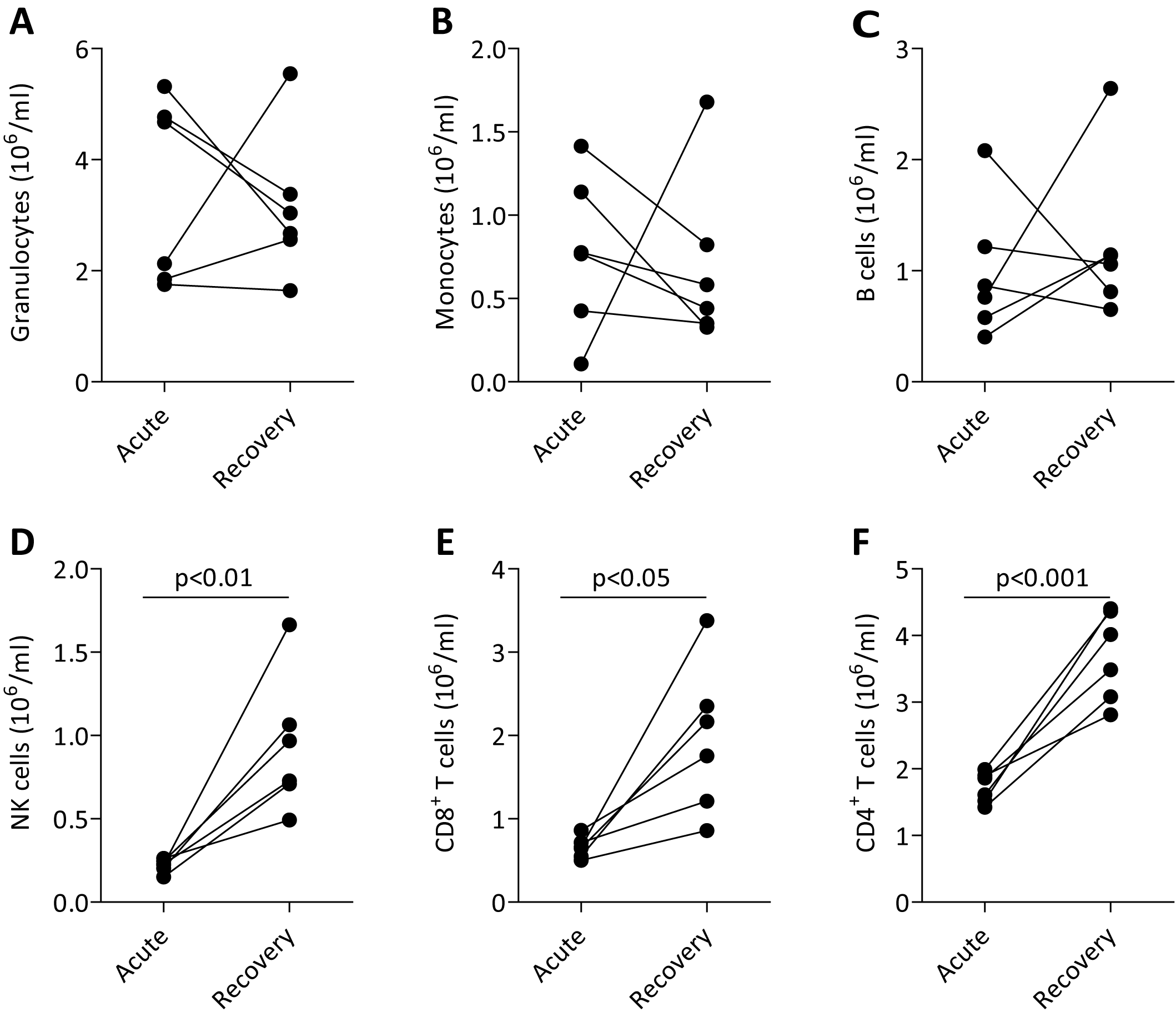

Supplement: S1 Fig — Immune phenotyping with flowcytometry of circulating leukocytes of infants with severe RSV infection (n = 6) in the acute phase and after clearance of the infection (on average 4 weeks after discharge). The numbers of NK cells as well as CD4+ and CD8+ T cells return to normal, indicating that the lymphopenia was transient. Statistics were performed by paired students-t test, significance was set at p<0.05. (TIF) [file pone.0131927.s001.tif]

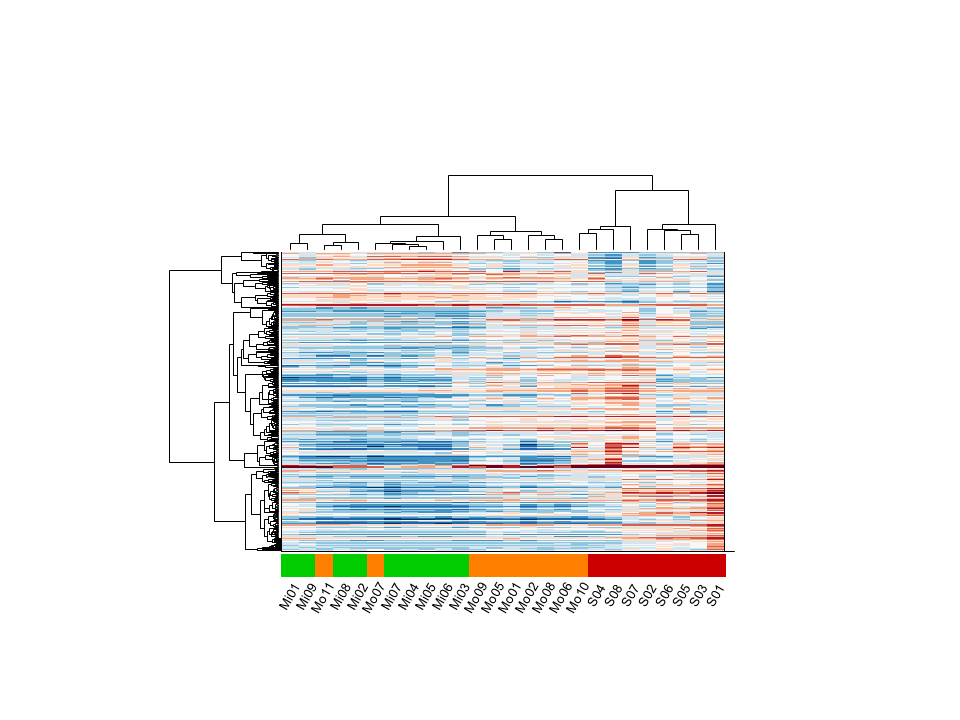

Supplement: S2 Fig — 448 differentially expressed probesets were selected based on overlap in the comparison mild vs severe disease in RSV infected children and acute samples vs recovery samples of children with severe RSV infection. Samples were clustered based on these selected probesets by complete linkage hierarchical clustering with 1-correlation as a distance measure. (TIF) [file pone.0131927.s002.tif]

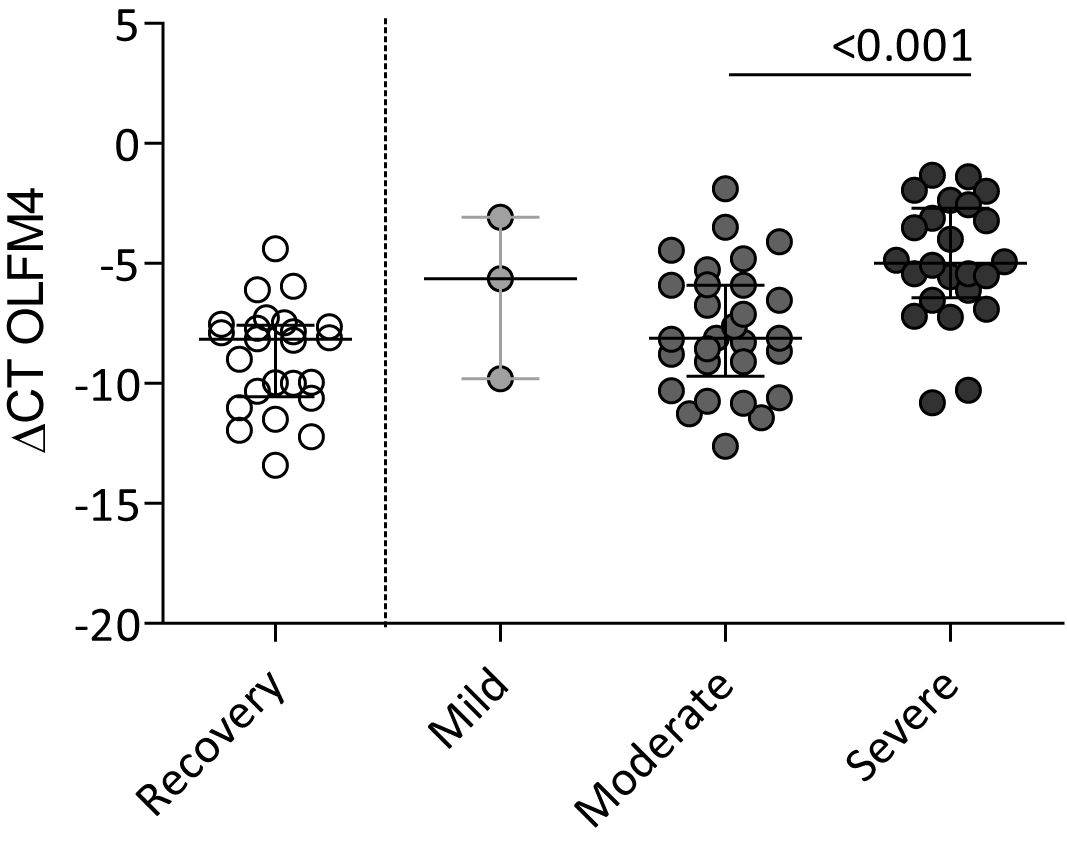

Supplement: S3 Fig — Expression levels are presented as ΔCt and median with inter quartile range (IQR). Statistics were performed by Kruskall Wallis tests (p<0.001), followed by Mann Whitney U tests for individual comparisons: mild vs moderate p = 0.36, moderate vs severe p<0.001, mild vs severe p = 0.51. (TIF) [file pone.0131927.s003.tif]
