# Supplementary material for: Olfactomedin 4 Serves as a Marker for Disease Severity in Pediatric Respiratory Syncytial Virus (RSV) Infection
Source: PLoS One. 2015 Jul 10;10(7):e0131927. doi: 10.1371/journal.pone.0131927 (PMC4498630; doi:10.1371/journal.pone.0131927)
Supplement: S1 Table — Values are given in numbers (percentages) and median and inter quartile range (IQR * mild versus severe p = 0.05, moderate versus severe p<0.001 ** mild versus moderate and severe p<0.001, moderate versus severe p<0.001 *** mild versus moderate p<0.01, mild versus severe p<0.05. (DOCX) [file pone.0131927.s004.docx]

**Supplemental table 1. Patient characteristics of validation cohort**

|  | **Mild**  **(N=14)** | **Moderate (N=42)** | **Severe**  **(N=24)** | ***p*-value** |
| --- | --- | --- | --- | --- |
| **Age (months)** | 3.2 [1.1-10.3] | 4.9 [2.0-14.5] | 1.2 [0.6-2.8] | *p*<0.01* |
| **Gender (male)** | 8 (57%) | 22 (52%) | 12 (50%) | NS |
| **Gestational age (wks)** | 36.8 [39.1-40.0] | 38.0 [37.0-40.0] | 38.9 [37.0-40.5] | NS |
| **Length of stay (days)** | 3 [2-3] | 6 [4-9] | 11 [10-13] | *p*<0.001** |
| **Confirmed RSV infection** | 3 (21%) | 29 (69%) | 15 (63%) | <0.01*** |

Values are given in numbers (percentages) and median and inter quartile range (IQR * mild versus severe *p*=0.05, moderate versus severe *p*<0.001 ** mild versus moderate and severe *p*<0.001, moderate versus severe *p*<0.001 *** mild versus moderate *p*<0.01, mild versus severe *p<*0.05.
